# Organ-Preserving Surgery for Penile Carcinoma

**DOI:** 10.1155/2008/634216

**Published:** 2008-11-04

**Authors:** Francisco E. Martins, Raul N. Rodrigues, Tomé M. Lopes

**Affiliations:** ^1^Departamento de Urologia, Centro Hospitalar Lisboa Norte, Hospital de Santa Maria, Universidade de Lisboa, Avenida Professor Egas Moniz, 1649-035 Lisboa, Portugal; ^2^Serviço de Urologia do Centro Hospitalar Lisboa Norte, Hospital de Santa Maria, Avenida Professor Egas Moniz, 1649-035 Lisboa, Portugal

## Abstract

*Introduction*. Penile carcinoma has traditionally been treated by either surgical amputation or radical radiotherapy, both associated with devastating anatomical, functional, and psychological impact on the patient's life. Innovative surgical techniques have focused on penile preservation in well-selected patients to minimize physical disfigurement and consequently maximize quality of life. The objective of this article is to define the current status of these organ-preserving surgical options for penile carcinoma. *Materials and Methods*. An extensive review of the Pubmed literature was performed to find articles discussing only reconstructive surgery which have contributed significantly to change traditional, frequently mutilating treatments, to develop less disfiguring surgery, and to improve patients' quality of life over the last two decades. *Results*. Several articles were included in this analysis in which a major contribution to the change in therapy was thought to have occurred and was documented as beneficial. Some articles reported novel techniques of less-mutilating surgery involving different forms of glans reconstruction with the use of flaps or grafts. The issue of safe surgical margins was also addressed. *Conclusion*. The development of less-disfiguring techniques allowing phallus preservation has reduced the negative impact on functional and cosmetic outcomes of amputation without sacrificing oncological objectives in appropriately selected patients based on stage, grade, and location of the tumour. Until more prospective studies are available and solid evidence is documented, organ preservation should be offered with caution.

## 1. INTRODUCTION

In the industrialized world, particularly in Europe and in the USA, penile carcinoma is an uncommon malignancy with an incidence of less than 1 per 100 000 of the male adult population. However, its incidence may be as high as 19 per 100 000 in parts of Asia, South America, Africa and may even reach 50 per 100 000 males in parts of north-eastern Brazilian states [1, 2]. This different worldwide distribution varies with age, circumcision, and hygiene patterns.

Historically, the majority (90%) are primary carcinomas, of which 95% are squamous cell carcinomas, involving the glans, prepuce, or both in over 78% of the cases. The management of penile carcinoma, particularly its invasive form, has changed little over the decades. Available treatments include surgical amputation and penis-preserving treatments, either surgical (circumcision, laser ablation, Mohs micrographic surgery, glansectomy associated with various forms of reconstruction) or nonsurgical (radiotherapy, immunotherapy, chemotherapy). Surgical amputation is the oldest of all modalities [3]. It has resulted in local control rates greater than 90% of the primary tumor and, therefore, remains the oncological “gold standard” for all stages [4–6]. Although the radical surgical approach provides excellent local control, it is often mutilating and is associated with urinary and sexual dysfunction as well as dramatic psychological morbidity [7]. These negative factors have led to a significant change in the approach to the primary penile lesion and to the development of several surgical organ-preserving techniques. Nowadays, the definitive treatment of penile carcinoma is stage-dependent, with the penile-preserving options especially reserved for low-grade and low-stage tumors. These techniques aim to remove as little of the functional anatomy as possible, without compromising local oncological radicality [8]. However, data from retrospective studies suggest a statistically higher local recurrence rate following penis-preserving treatments compared with radical surgery. Most recurrences are surgically salvageable and overall mortality is comparable to primary amputation [9]. The objective of this article is to give an overview of the current status and the role of these organ-preserving surgical options for penile carcinoma and state their limitations.

## 2. INDICATIONS AND GOALS OF ORGAN PRESERVATION

Some retrospective studies have reported good cosmetic and functional outcomes with conservative treatment options and an overall organ preservation of 60% [9]. Because about 80% of penile carcinomas occur distally, involving the glans and/or prepuce, they are potentially amenable to organ-preserving surgery [10, 11].

It is generally accepted that patients with penile carcinomas associated with favourable histology (stages Tis, Ta, T1; grades 1 and 2) are at low risk for local progression and/or distant metastatic spread (Table 1). These patients are also the best candidates for penile-/glans-preserving treatment options [2]. Recently, however, some series have suggested that these indications can be expanded in order to include T2 and even some distal T3 tumors as well as recurrences after radiotherapy [8]. Nonetheless, until more rigorous scientific evidence is available, organ-preserving strategies should be reserved to well-selected patients with limited low-grade, low-stage disease [11, 12]. A traditional 2 cm excision margin has been challenged as unnecessary for patients undergoing partial penectomy for squamous cell carcinoma. Conservative techniques involving surgical margins of only less than 10 mm appear to offer excellent oncological control [13, 14].

The goals of penile-preserving treatments are to maintain penile/glans sensation and to maximize penile shaft length where possible. However, cosmetic and functional results should not compromise long-term oncological outcomes.

## 3. METHODS OF SURGICAL ABLATION FOR ORGAN PRESERVATION

A variety of penile-preserving therapeutic approaches have been used for low-grade and low-stage penile carcinoma, including topical treatments (5-fluorouracil or imiquimod cream for Tis only), radiotherapy, Mohs micrographic surgery, laser ablation or excision, and conservative excision strategies (Table 2). This article will focus exclusively on surgical strategies to achieve organ preservation. Nonsurgical options are beyond the scope of this review.

### 3.1. Mohs micrographic surgery

Mohs micrographic surgery (MMS) refers to a surgical technique of excising accessible tumors under microscopic control [15]. The tumor is excised in layers and the undersurface of each layer is examined microscopically by systematic frozen sections in multiple sessions. This excision is continued until the undersurface of the excised tissue is negative, at which point another section of tissue is removed to ensure a clear resection margin. This sequential microscopic guidance offers increased precision and control of the negative surgical margin, while maximizing safe organ preservation. MMS is most commonly used for skin tumors but the accessibility of penile carcinomas (most commonly on the glans) makes it a suitable candidate for such a procedure. In Mohs' 50-year experience with 35 cases, the success rate was stage-dependent. A percentage of 86% of stage T1 and 82% of stage T2 cases were tumor-free compared to none of stage T3 at a followup of 5 years.

This technique is attractive because it allows reassurance of local complete excision and preservation of local penile anatomy and function. However, because local failure rate is apparently higher (32%) than amputation, it should be reserved to patients with penile carcinoma in situ or with small, distal, superficially invasive tumors. Further reports with this technique are necessary to allow comparison and reproducibility of outcomes in order to encourage its more widespread use. Complications may include meatal stenosis and glans disfigurement.

### 3.2. Laser ablation or excision

Penile laser surgery has been used since the 1980s. The four types of lasers used are carbon dioxide, argon, neodymium yttrium aluminium garnet (Nd:YAG), and potassium titanyl phosphate (KTP) lasers, the CO_2_ and Nd:YAG modes being the most commonly used in current practice [16, 17]. CO_2_ laser has a very low penetration power (only 0.1 mm) and is, therefore, unsuitable for most tumors, resulting in recurrence rates of up to 50% [18]. Nd:YAG has a much higher penetration power of about 6 mm due to its rather short wavelength (*λ* = 1.06 *μ*m, i.e., 10 times less than CO_2_), resulting in protein denaturation at such depth. Overall recurrence rates after laser ablation are also stage-dependent, averaging 7.7% for Tis tumors, and as high as 25% for T1 lesions [16]. Other authors have reported good outcomes after Nd:YAG laser for T1 tumors with excellent cosmetic and functional results and high satisfaction rates. Recurrences were noted in 6.9% of the patients, which is comparable to recurrence rates after partial amputation (0–8%) [19].

The available data to date demonstrate that laser surgery is feasible and may achieve results comparable to those of traditional amputative surgery, particularly in highly selected patients and in conjunction with frozen-section biopsies. Additionally, it has significant anatomical, cosmetic, and functional advantages over traditional amputation. However, as the local recurrence is higher, a close surveillance is mandatory for early detection. Therefore, patient selection is extremely important. Because in laser surgery the depth of tumor invasion is crucial, only those invading less than 6 mm into tissues are suitable for this treatment modality.

### 3.3. Conservative surgery

**Circumcision**

It is the most simple and common surgical procedure in the management of penile carcinoma. The majority of men with penile carcinoma are uncircumcised. It is indicated for symptomatic treatment of painful or haemorrhagic tumors as well as for acquired phimosis secondary to preputial tumors. It is always recommended before radiotherapy as it allows better targeting and definition of the tumor, simultaneously preventing preputial radiotherapy-related adverse reactions, and, above all, it improves local oncological surveillance. Noteworthy, circumcision alone is a sufficient primary curative treatment for small low-stage (Tis, Ta, T1) and low-grade (grades 1 and 2) disease limited to the distal prepuce [20]. If the tumor is more proximal and close to the coronal sulcus, the circumcision margin will need to be extended proximally to the penile shaft to ensure adequate oncological resection, as recurrence rates may be as high as 50% [21]. Therefore, case selection is critical to reduce local recurrence rates.

**Glansectomy**

It can be done either partial or total, has recently been introduced for the local excision of distal tumors on the glans and prepuce [8, 12, 22, 23]. Frozen sections from the cavernosal bed and urethral stump should be carried out during the procedure to ensure negative surgical margins followed by an end-shaft urethrostomy. Glansectomy is usually combined with grafting procedures to create a neoglans. Basically, there are 2 forms of glansectomy: (i) partial glansectomy, which removes the portion of the glans affected by the tumor, leaving behind remaining glanular epithelium with malignant potential, and (ii) total glansectomy, which removes all the glans tissue, thus preventing ‘de novo’ tumor growth.

**Figure 1:**
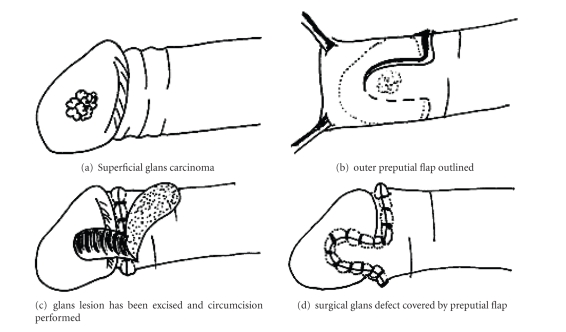
Outer preputial full-thickness skin flap as described by Ubrig et al. (2001) to cover surgical glans defects.

Traditionally, amputative surgery has been based on the assumption that a 2 cm resection margin is required to achieve local oncological clearance [24]. However, the scientific value of a 2 cm margin has not been supported uniformly and several authors have recently questioned it [13, 14, 25], concluding in their studies that a 2 cm surgical margin was not only unnecessary but also overtreatment in many cases. About 80% of the penile carcinomas arise distally, which render them potential candidates for penile-preserving surgery. This type of surgery includes an extirpative component leaving in some cases a simple defect amenable to primary closure. If the defect is larger and primary closure is not possible or safe, various techniques have been suggested to cover or reconstruct the area [8, 12, 22, 23, 26–33]. Ubrig described a simple technique in 2001 in which an outer preputial skin flap was used to cover the glans defect if primary closure was impossible. However, the tumor should not be too deep (Figure 1). Pietrzak et al. have suggested the use of a full-thickness flap of penile skin or extragenital (lateral aspect of the thigh) split-thickness skin graft to reconstruct the glans associated with partial or total glans removal. In cases of invasion of tunica albuginea by distal tumors, distal corporectomy was included [8]. In glans-preserving procedures, partial glansectomy with primary glans closure was essentially an excisional biopsy of a small distal tumor. Larger lesions necessitated partial glansectomy followed by glans reconstruction which was performed with the use of split-thickness or full-thickness grafting. In glans-removing procedures, total glansectomy was performed followed by either split-thickness skin graft reconstruction or reconstruction of cavernosal tips and grafting, if a distal corporectomy was required. In some cases, a penile-lengthening procedure was added to the reconstruction to maintain as much cavernosal tissue as possible. In all forms of penile-preserving surgery, a frozen biopsy of the surgical bed is mandatory to confirm tumor clearance (negative margins). A subtotal glans excision without grafting has been described as a simple and cosmetically attractive alternative to other forms of conservative surgery for penile carcinoma [23]. This procedure involves excision of the tumor and glans between 2 incision lines leaving the urethra intact. The residual glans and urethral meatus is sutured down to the distal corpora and the penile skin is advanced to be sutured to the distal glans at the level of spatulated urethra. A urethral catheter is left indwelling for 24 hours. No skin or any other source of grafting is required and the patient is discharged the next day. Apparently, patients maintain their voiding characteristics unchanged (e.g., no spraying) and avoid graft-related complications (e.g., donor-site morbidity, graft failure, and infection). However, this technique should be avoided in patients with penile tumors very close to (less than 5 mm) or invading the urethral meatus. Other forms of glansectomy without glans reconstruction have also been described [22, 33]. These usually create a new urethral stoma and attach the residual urethra to the foreskin with acceptable cosmetic and functional outcomes. However, some authors have reported that these procedures only partially resolve aesthetic and psychological problems associated with surgery. Also, they do not resolve the question of penile sensation, and consequently ejaculation and orgasm, as well as penile length and appearance. To overcome these pitfalls, they suggested a technique of glans reconstruction using the distal urethra [27].

**Figure 2:**
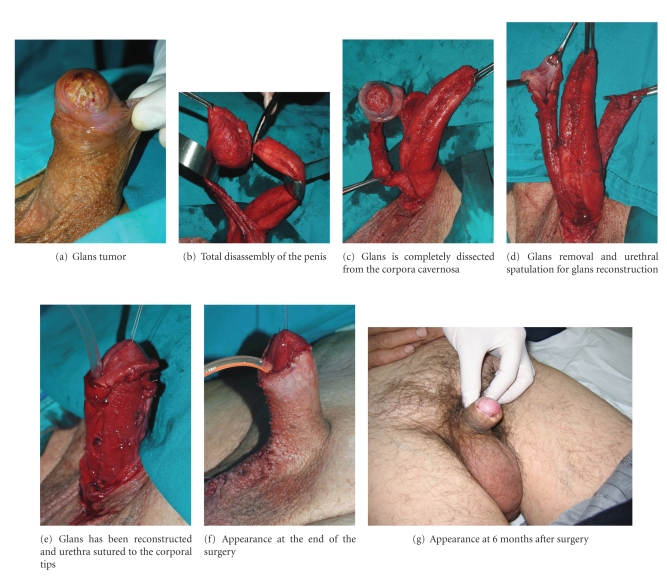
Penile disassembly for the conservative treatment of penile carcinoma.

More recently, an alternative approach to organ-sparing surgery for penile carcinoma based on a penile disassembly technique has been utilized by Djordjevic et al. with good results [28]. Penile disassembly was first described by Perovic in the early 1990s as a surgical technique to treat most congenital and acquired penile deformities in paediatric and adult male populations, such as hypospadias, extrophy-epispadias complex, penile curvature, and Peyronie's disease. This technique has been employed in low-grade and low-stage tumors, mostly T1G1-2 lesions. The procedure begins with urethral mobilization together with Bucks fascia (Figure 2). Dorsally, the neurovascular bundle is dissected off by blunt and sharp manoeuvres. Glans with urethra ventrally and neurovascular bundle dorsally are completely separated from the corpora cavernosa. The neurovascular bundle is divided 2 cm proximal to the glans cap. The glans is removed after division of the urethra. Biopsies of the surgical margins are performed routinely to confirm oncological clearance. The urethra is spatulated 4 cm in length and sutured to the corpora cavernosa. The spatulated urethra is used for neoglans construction. The corpora cavernosa are fixed to the skin proximally using U-shaped sutures to avoid penile retraction. Reconstruction of the penile skin is performed as in circumcision. The authors believe that penile disassembly represents a radical but very useful approach to organ-preserving surgery in penile carcinoma with excellent cosmetic, functional, and oncological outcomes.

Some authors have long reported on a surgical strategy for refashioning of phallus stumps to make them longer and more natural in appearance. Where this was not feasible, a neophallus was performed. Perineal urethrostomy was avoided completely [29, 33]. This was even considered to reflect a failure of surgical skill [29].

Total glansectomy for penile tumors was first described by Austoni in 1996 [34]. Since that time, enormous efforts have been made in the development of more refined and appealing surgical alternatives to improve both function and cosmesis, as well as local oncological control. Early results have been encouraging but more reproducible studies and longer followup are still required to consider organ preservation as the gold standard treatment of penile carcinoma. Until then, it should be used with caution.

## 4. CONCLUSION

Historically, amputative surgery and radical radiotherapy were the only options to treat penile carcinoma. Over the last two decades, several innovative techniques have been described and proposed for organ-preserving surgery in penile carcinoma. These should avoid complications and maximize both cosmetic and functional outcomes, simultaneously not compromising local oncological long-term control. At present, definitive management of penile carcinoma remains stage-dependent. Penile amputation has been challenged by more recent conservative surgical techniques and should perhaps be considered overtreatment in low-stage disease. Glansectomy appears to offer good local control rates. Glans reconstruction with or without grafting procedures have offered excellent cosmetic results where applicable. However, until further studies are available and sufficient evidence reproducible in common day practice, and until the surgical margin issue is safely addressed in prospective studies, penile amputation (partial or total) with all its attendant anatomical, psychosocial, and sexual disabilities should still be regarded as the gold standard treatment for all stages of penile carcinoma, even for Tis, Ta and T1 tumors.

## Figures and Tables

**Table 1:** TNM classification of penile carcinoma (1997/2002).

T-Primary tumor
TX Primary tumor cannot be assessed
T0 No evidence of primary tumor
Tis Carcinoma *in situ*
Ta Non-invasive verrucous carcinoma
T1 Tumor invades subepithelial connective tissue
T2 Tumor invades corpus spongiosum or cavernosum
T3 Tumor invades urethra or prostate
T4 Tumor invades other adjacent structures

N-Regional lymph nodes

NX Regional lymph nodes cannot be assessed
N0 No evidence of lymph node metastasis
N1 Metastasis in a single inguinal lymph node
N2 Metastasis in multiple or bilateral superficial lymph nodes
N3 Metastasis in deep inguinal or pelvic lymph nodes, unilateral or bilateral

M-Distant metastasis

MX Distant metastases cannot be assessed
M0 No evidence of distant metastases
M1 Distant metastases

**Table 2:** Organ-preserving therapeutic strategies for penile carcinoma.

A nonsurgical					
(1)	Topical treatments	5-Fluoroacil solution			
		Imiquimol cream			

(2)	Radiotherapy	Plesiotherapy			
		Interstitial brachytherapy			
		External beam radiotherapy			

(3)	Cryosurgery				

(4)	Chemotherapy				

(5)	Immunotherapy				

B Surgical					

(1)	Laser ablation or excision	CO_2_			
		Nd:YAG			
		KTP			

(2)	Mohs micrographic surgery				

		Circumcision			
(3)	Conservative surgery	Glans-preserving techniques	Partial glansectomy	with primary closure	
				with graft reconstruction of the glans	Split-thickness skin grafts
					Full-thickness skin grafts
					Buccal mucosa
		Glans-removing techniques	Total Glansectomy	with split-thickness skin grafts	
				with distal corporectomy and reconstruction	
